# Recruiting and retaining participants in three randomised controlled trials of psychological interventions conducted on acute psychiatric wards: top ten tips for success

**DOI:** 10.1192/bjo.2022.527

**Published:** 2022-07-07

**Authors:** Pamela Jacobsen, Gillian Haddock, Jessica Raphael, Craig Peak, Rachel Winter, Katherine Berry

**Affiliations:** Department of Psychology, University of Bath, UK; and Department of Psychology, Institute of Psychiatry, Psychology, and Neuroscience, King's College London, UK; Division of Psychology and Mental Health, School of Health Sciences, University of Manchester, UK; Manchester Academic Health Sciences Centre, UK; and Greater Manchester Mental Health NHS Foundation Trust, UK; Greater Manchester Mental Health NHS Foundation Trust, UK; Greater Manchester Mental Health NHS Foundation Trust, UK; Greater Manchester Mental Health NHS Foundation Trust, UK; Division of Psychology and Mental Health, School of Health Sciences, University of Manchester, UK; Manchester Academic Health Sciences Centre, UK; and Greater Manchester Mental Health NHS Foundation Trust, UK

**Keywords:** Psychiatric hospitals, randomised controlled trial, patient recruitment, individual psychotherapy, clinical psychology

## Abstract

**Background:**

It is essential to conduct randomised controlled trials of psychological interventions on acute psychiatric wards to build a robust evidence base for clinical practice.

**Aims:**

This paper aims to share strategies from three different in-patient trials that successfully recruited and retained participants, to disseminate good practice for the conduct of future trials in this challenging and complex clinical setting.

**Method:**

We present strategies from three in-patient trials of psychological interventions: TULIPS (Talk, Understand, Listen for Inpatient Settings), amBITION (Brief Talking Therapies on Wards) and INSITE (Inpatient Suicide Intervention and Therapy Evaluation). All studies recruited participants from acute in-patient wards, initiated therapy within the in-patient setting and followed up on participants post-discharge.

**Results:**

We summarise our recommendations for good practice in the form of ten top tips for success, based on our collective experience of conducting trials on psychiatric wards. Key themes relate to the importance of relationships between the research team and clinical staff; good stakeholder involvement and getting early buy-in from the team; and adapting to the particular demands of the clinical setting.

**Conclusions:**

Sharing good practice recommendations can help reduce research waste arising from poor recruitment and/or retention in future in-patient clinical trials.

## Recruitment and retention in clinical trials

Recruitment and retention are two of the biggest challenges in conducting randomised controlled trials (RCTs). For example, a recent review of RCTs funded by the UK's National Institute for Health Research (NIHR) found that recruitment and retention varied widely across trials (*N* = 151), with only 56% of trials recruiting to their pre-specified target sample size.^1^ Problems with low recruitment and retention are significant as they result in a loss of statistical power, unrepresentative samples of participants, biased follow-up data in the case of participant attrition and ultimately, a waste of funders’ money and participants’ time. These challenges are especially prevalent in conducting research with people with severe mental health difficulties, who are often considered a particularly difficult clinical population to recruit and retain in trials.

Systematic reviews^2–4^ have identified barriers to trial recruitment, which include issues related to funding, design, recruiter and participant. Studies focused on barriers to recruiting to trials involving people with severe mental health diagnoses highlight the relevance of factors that are apparent in RCTs in general. These include misconceptions about the different groups in the trial (trial arms), lack of equipoise, variable interpretations of eligibility criteria and lack of staff time and resources to support recruitment. There are also additional factors that are unique to recruiting participants with mental health diagnoses. For example, staff paternalism can lead to excessive gate-keeping, and poor staff–patient relationships may make staff reluctant to approach potentially eligible participants about research opportunities.^5–7^

## Particular challenges of in-patient trials

Previous reviews demonstrate the benefits of psychological therapies for severe mental health problems, in terms of improvements in symptoms and reduced costs to the National Health Service (NHS).^8,9^ However, recent systematic reviews have found that very few controlled trials of psychological therapies target psychiatric wards.^10–12^ Although there is good evidence for psychological therapies from high-quality trials with out-patients, existing evidenced-based psychological therapies need to be adapted and then trialled within in-patient settings. The process of delivering therapy in in-patient settings is likely to present unique challenges that require empirical investigation. For example, in-patients are likely to be experiencing higher levels of distress, may be at high-risk of self-harm/suicide,^13^ and the relatively short length of stay (average 31 days in UK)^14^ may affect the potential length of therapy. There are also many environmental factors to consider, such as noise and disruption on the ward,^15,16^ a lack of appropriate spaces in which to conduct therapy^17^ and interruptions to therapy sessions.^18^

There is a perception of general reluctance to conducting RCTs on in-patient wards, given that it is often viewed as a chaotic and uncontrollable environment, with in-patients being frequently admitted and discharged. There is also an increased need to consider attitudes of in-patient staff toward the provision of therapy in this setting. For example, staff can be concerned that it's not ‘the right time’ to offer therapy, or that admissions are not long enough to deliver any kind of meaningful therapy.^19^

It is clearly important to overcome these barriers to conducting in-patient trials, given that there is an urgent need to increase access to evidence-based psychological therapies in these settings. On the back of significant lobbying by patient and carer groups, the Mental Health Task Force (2016) now advocates a ‘referral to treatment access’ standard for psychological therapy in acute in-patient care, meaning that NHS mental health trusts will need to deliver timely, evidence-based psychological treatments in these settings.^20^

The aim of the current paper is to describe strategies that we believe have helped us to achieve good recruitment and retention in these trials, with the aim of helping others carrying out trials with similar populations in similar settings.

## Method

In this section we present three case studies of trials conducted on NHS psychiatric in-patient wards in the UK, to provide the wider context for our top tips for recruitment and retention for in-patient trials. We chose these three trials based on the senior authors (P.J., K.B., G.H.) experiences of conducting in-patient trials, and as exemplars of in-patient trials that had high recruitment and retention rates.

The TULIPS trial (Talk, Understand, Listen for Inpatient Settings) is a cluster-randomised RCT currently open to recruitment (prospectively registered on Clinicaltrials.gov under identifier NCT039503882, registered on 15 May 2019). The aim of the study is to randomise 34 wards in psychiatric hospitals across the UK to receive either treatment as usual (TAU) or a newly appointed half-time senior clinical psychologist over a 7-month period. The intervention being evaluated is a stepped model of care delivered by the psychologist, including direct therapeutic work with in-patients and indirect work with ward staff. Key outcome measures for the trial are ward level incidents of violence, aggression and self-harm, and self-reported in-patient well-being, social functioning and service use, staff burnout and both staff and in-patient perceptions of ward atmosphere. Both staff and in-patients are required to complete outcome measures at baseline, 6-month and 9-month follow-ups, with the majority of in-patients being followed up in community settings post-discharge. Inclusion criteria for both staff and in-patients are capacity to consent and able to complete self-report measures. In-patients are potentially eligible for participation regardless of diagnosis or main psychiatric symptoms. The trial is ongoing, with staff and in-patient recruitment rates currently on track for at least 80% of the prespecified target.

The amBITION trial^21^ is a completed feasibility RCT of a brief therapy for psychosis, which was conducted on four wards within a psychiatric hospital in London, UK (prospectively registered on the ISRCTN Registry under identifier ISRCTN37625384, registered on 19 August 2015). In addition to TAU, participants were randomly allocated to receive either mindfulness-based crisis intervention or a control intervention (social activity therapy), for one to five sessions. All sessions followed a stand-alone, self-contained format, to accommodate unpredictable lengths of stay and unexpected discharges. The treatment phase was restricted to the duration of the in-patient admission (maximum 5 weeks). Therapy was delivered by a single trial therapist, who was not part of the usual ward team. Primary outcomes were feasibility of recruitment and retention, and secondary clinical outcomes were readmission to hospital, and self-report measures of psychotic and affective symptoms. Measures were taken at baseline, discharge and 3- and 6-month follow-up. Inclusion criteria were people newly admitted to in-patient care, presenting with positive symptoms of psychosis, in the context of a schizophrenia spectrum or affective disorder, and having capacity to consent to participate. Fifty participants were recruited (83% of target sample size of 60), and follow-up rates at 6 months post-discharge were 98% for service use data extracted from clinical notes, and 86% for self-report questionnaire measures. Recruitment and retention rates both exceeded pre-set feasibility benchmarks.^22^

The INSITE trial (Inpatient Suicide Intervention and Therapy Evaluation)^23^ is a completed feasibility and acceptability RCT of a psychological therapy for in-patients who are suicidal, which was conducted on wards within a NHS trust in the North-West UK (retrospectively registered on the ISRCTN Registry under ISRCTN17891026, registered on 22 April 2015). In-patients were randomly allocated to receive either cognitive–behavioural suicide prevention in addition to TAU, or TAU alone. The intervention consisted of TAU plus up to 20 cognitive–behavioural suicide prevention sessions, delivered over 6 months by experienced therapists who were not part of the usual ward team. The intervention began in the in-patient setting but continued into the community if a participant was discharged before they had completed treatment. Primary outcomes were feasibility and acceptability of the intervention, and secondary outcome measures included suicidal thinking, behaviours, functioning, quality of life and service use. Measures were taken at baseline, 6 weeks and 6 months (end of treatment). Inclusion criteria were in-patients experiencing suicidal thoughts or behaviours within the 3 months before admission (any diagnosis), and having capacity to consent to participate. Fifty-one participants were recruited (85% of target sample size of 60), and follow-up rates at both 6-week and 6-month follow-up were 73%, which was slightly lower than the anticipated 80% retention rate.^24^

### Ethics approval and consent to participate

The authors assert that all procedures contributing to this work comply with the ethical standards of the relevant national and institutional committees on human experimentation and with the Helsinki Declaration of 1975, as revised in 2008. All procedures involving human patients were approved by the following areas: North West – Greater Manchester East Research Ethics Committee (approval number: 19/NW/0316; TULIPS), London – Camberwell St Giles Research Ethics Committee (approval number: 15/LO/1338; amBITION) and NRES Committee North West – Lancaster (REC reference number: 13/NW/0504; INSITE). All participants gave written consent.

## Results

### Successful recruitment and retention: ten top tips for success

The first priority should always be to build, and maintain, a strong relationship with all members of the multidisciplinary ward team. Establishing support from senior management (e.g. ward manager and overall service managers) is necessary, but not sufficient if it is not combined with buy-in from the overall ward team (including healthcare assistants, nurses, occupational therapists, psychiatrists and administrative staff) who will be involved in the trial on a more day-to-day basis.

The second tip is that the likelihood of getting strong buy-in from the team is greatly increased if the ward staff have been consulted and involved in the design of the trial and intervention, rather than feeling like it has been imposed on them, if this is possible. To further enhance staff engagement, using terminology such as ‘joint NHS and university project’ can be helpful, as opposed to ‘research trial’, which can be perceived as external to the usual work of the ward.

The third tip is that ward teams are always busy, and they have many competing demands on their time and attention, so make it as easy as possible for them to remember the trial. It is advisable to gather information from the ward manager about the best times to visit the ward, noting down the ward routine (e.g. ward round days).

The fourth tip is that relying solely on busy ward staff to regularly screen and approach in-patients will most likely lead to poor recruitment, and potentially eligible participants will be missed. It is therefore best to ensure that proactive screening of new referrals for the trial is done by research staff, fitting in with usual ward routines and liaising regularly with ward staff.

The fifth tip is that research staff must be prepared to repeat information about inclusion and exclusion criteria on a regular basis, as ward teams can have a high turnover. Conversations in person can be supplemented with simple information sheets tailored specifically for staff, but reliance on written material only should be avoided.

The six tip is that it is important to listen to staff and respect their views about who to approach about the study. However, this needs to be balanced with the need to protect against ‘gate-keeping’, which might inadvertently prevent potentially eligible people from being offered the chance to participate.

The seventh tip is that wards can be chaotic environments, and sometimes in-patients may be taking leave off the ward, may be asleep, with other visitors or not feel up to meeting when research staff have arranged to meet with them. However, it is essential that research staff build a reputation for reliability by always turning up at the agreed time and date for all appointments.

The eighth tip is to randomise participants as close to the start of treatment as possible. Even a 24 h window can lead to losing participants between randomisation and start of treatment because of unplanned discharges or other unforeseen factors.

The ninth tip is that to successfully follow people up after they leave hospital, it is important to be flexible, prepared and willing to go the extra mile. People often change their mobile numbers, sometimes do not have battery or credit, or do not like answering the phone. Therefore, aim to get as many different types of contacts for a person as possible, and seek permission to contact via a trusted third party where appropriate.

The tenth and final tip is that doing follow-up assessments at the same time as routine clinical appointments, such as when people meet with their care coordinator, can be very helpful to save the need for multiple trips. Not all participants will be happy to meet in a healthcare setting – in this case, be flexible and offer alternatives such as home visits (visiting in pairs, or with members of the community team is recommended if there are unknown risk to researchers). Building up a good working relationship with community teams will help facilitate this joint working.

See Table 1 for examples of these approaches across the three case study trials.

**Table 1:** Summary of top ten tips for successful recruitment and retention

		Illustrative examples	
	TULIPS trial	amBITION trial	INSITE trial
Relationship with ward team	- Researchers meet with all ward managers before the start of recruitment to discuss and address any concerns - Research assistants based on ward during recruitment period to help them get to know the team better - Research assistants adhered to safety protocols at all times, e.g. wearing alarms on the ward. Therapy sessions were documented in the clinical notes in a timely manner, including noting of any risk issues	- Researcher met with all ward managers and consultant psychiatrists individually before recruitment started - Researcher attended team meetings with staff regularly and spent time in the nursing office to allow opportunities for informal contact - Researcher followed standard ward protocols, e.g. checking in with the nurse in charge on arrival on the ward for a risk briefing, and feeding back any risk issues to nursing staff straight away	- Research assistants based on the ward during recruitment, where possible and had frequent meetings and regular communication with ward team about the trial - Research assistants adhered to ward protocols at all times, including following protocols for reporting risk issues
Involve ward staff in design of trial where possible	- Phase 1 of the project (18 months) was dedicated to designing the trial with ward staff and local clinicians as key stakeholders (*N* = 60), including interviews and expert consensus studies. A study champion identified on each ward once study started to promote the trial within the staff team	- In-patient psychologists and ward managers were consulted in the design of the trial to ensure feasibility	- Phase 1 involved qualitative interviews with in-patient staff (*n* = 20) and in-patients (*n* = 20) to inform the development of the treatment protocol and trial design
Remember ward teams are busy	- Study promotional materials (e.g. pens, mugs) were left on the ward as reminders for the times when research staff were not present (e.g. on night shifts)	- Researcher fit in with existing meeting structures to liaise with staff, e.g. handovers	- Trial protocol was designed as to not place undue burden on staff team, or create extra work (e.g. ward staff were not expected to speak to all new eligible admissions about the trial, this role was taken by research assistants and staff from the local Clinical Research Network)
Do not rely solely on ward team to screen for participants	- Because of a narrow recruitment window, research assistants? discussed potentially eligible with the ward team on a daily basis	- Screening for participants was discussed by researcher at handover meetings at least twice a week	- Researchers did daily (where possible) screening of new admissions at high risk for self-harm/suicide. Potentially eligible in-patients were not always approached immediately, as people sometimes preferred to have a few days to settle on the ward before being approached about therapy
Repeat information about inclusions/exclusion criteria	- Ward staff and in-patients were involved in preparing trial literature, and advising how these could best be displayed and communicated	- Researcher repeated inclusion/exclusion criteria for trial frequently when discussing potentially eligible participants - There was a staff version of the trial information sheet, which was circulated regularly	- Phase 1 of the project included development of inclusion/exclusion criteria that were viewed as clear and appropriate by ward staff
Be aware of gatekeeping issues around approaching in-patients to take part in trial	- Research assistants explored staff views about why they thought a person would/would not be likely to engage, and therefore who should be approached, in a collaborative way to help build a shared understanding of the trial procedures (i.e. that all eligible in-patients should be approached, even if staff judged them unlikely to accept offer of therapy)	- Trial protocol stated that all potentially eligible in-patients (accounting for capacity/risk issues) should be approached and offered information about the trial, regardless of how likely they were seen to accept the offer	- All trial information/literature presented as to encourage ‘equipoise’ in the ward team, to ensure that randomisation to TAU not seen as a negative outcome for in-patients who staff perceived as having a high need for therapy.
Adjust to the chaos without being chaotic	- Research assistants were flexible in their working hours, including offering to see in-patients in the evening when wards are less busy	- Participants were given appointment cards with the time/date of the next appointment, and this was also recorded on their electronic health record so it could be shared with the ward team	- Research assistants were flexible in offering appointments for baseline and follow-up assessments, and therapy appointments (one to two per week) were also scheduled flexibly
Minimise time window between randomisation and start of therapy	- Cluster randomisation (unit of randomisation was the ward), with intervention on wards started immediately after baseline assessments completed	- Participants were randomised at the start of their first treatment appointment, using an online randomisation portal from the Clinical Trials Unit, accessed via a laptop on the ward. This was successful in preventing any participant drop-out between randomisation and start of therapy in the trial	- Participants were randomised as close to start of treatment as possible, after completing baseline assessments. The majority of participants received at least one therapy session (mean of 11 sessions out of a maximum of 20)
Be flexible and persistent with post-discharge follow-ups	- As many kinds of contact details were collected from participants as possible, including mobile/landline numbers and e-mail/postal addresses. Research assistants checked that contact details were still valid a month in advance of when follow-ups were due, to give time to update details where necessary	- Where participants did not use or disliked being contacted by phone, permission was sought to arrange follow-up via a third party, such as a relative or keyworker from their CMHT	- Trial end-point was 6 months after baseline assessment, but therapy sessions could continue into the community after discharge up to a 6-month treatment window, increasing the likelihood of the research team maintaining contact with the participant after discharge
Offer follow-up assessments at the same time as routine clinical appointments	- Participants given options as to when and where to schedule follow-up assessments	- Follow-up appointments were offered at CMHTs/hospital out-patient department, before or after participants attended routine care appointments, to save multiple trips	- Participants given options as to when and where to schedule follow-up assessments

TULIPS, Talk, Understand, Listen for Inpatient Settings; amBITION, Brief Talking Therapies on Wards; INSITE, Inpatient Suicide Intervention and Therapy Evaluation; TAU, treatment as usual; CMHT, community mental health team.

## Discussion

Recruiting to target, and retaining participants at follow-up, is challenging but possible for RCTs of psychological interventions conducted on psychiatric wards. This paper aimed to share strategies from three different in-patient trials that successfully recruited and retained participants, to disseminate good practice for the benefit of other researchers planning future research in similar areas.

The themes running through our tips primarily relate to the importance of relationships between the research team and clinical staff; good stakeholder involvement and getting early buy-in from the team; working effectively with ward staff who take on gatekeeper roles; and adapting to the particular demands of the clinical setting. Our findings are consistent with previous literature on successful recruitment to trials involving people with severe mental health problems that have also emphasised the key importance of the relationship between researchers and recruiting clinicians,^25^ and support from senior staff to encourage staff participation.^26^ A large-scale survey of clinical studies officers in mental health research networks (*N* = 170) found that a positive attitude toward the study among gatekeepers was the most commonly endorsed factor that was seen as facilitating trial recruitment. Conversely, gatekeeper perceptions that the intervention being tested would not likely be effective or appropriate for the target population was one of the most commonly reported barriers to recruitment, as it was seen as leading to staff being paternalistic and overprotective.^27^ This is particularly relevant to recruitment of clinical populations such as people receiving in-patient psychiatric care, as staff may hold unhelpful beliefs about psychological interventions not being helpful, or potentially being destabilising at this point in the care pathway.^19^ Similarly, a previous study has found that mental health nurses can be reluctant to refer people with psychosis to take part in trials if they think they would decline to participate, or may lack capacity to consent, even if this is not the case.^28^ However, previous research shows that patients in general appreciate the opportunity to be invited to take part in research trials because they would like to help others with a similar condition, and to advance scientific knowledge in general,^29,30^ and this has also been found to be the case for people diagnosed with severe mental health problems.^31^ Talking to staff about their concerns and providing relevant counter-information can help allay such concerns.

Working in partnership with key stakeholders, including patients, carers and families, service managers and front-line clinical staff, is crucial for successful recruitment. Embedding these processes early on the process, and maintaining them throughout, is important as service managers and staff can feel reluctant to refer people into a trial they feel was approved ‘above their heads’.^6^ This approach also helps to overcome any prior pessimism from staff regarding the likelihood of adopting research into practice following the completion of the research period, based on previous experiences of research.^32^ Theories from the field of implementation science, such as normalisation process theory,^33^ similarly emphasise the importance of staff ‘buy-in’ when implementing any new practice into a complex system, so that they can make sense of what they are being asked to do, and integrate it smoothly with existing practice. Relationships need to be built interpersonally – winning ‘hearts then minds’, as Patterson and colleagues put it.^34^ Relationships can be built effectively by establishing common ground between the values and intentions of the research team, and the clinical team (e.g. improving access to effective therapies).

Adapting to the fast-paced and often unpredictable nature of acute psychiatric care is also essential. We found that tailoring the trial design to the clinical setting, such as having more frequent screening visits to the wards and minimising the time window between randomisation and start of treatment, were crucial to success. These are analogous to adaptations already used in clinical trials in other challenging settings, such as hospital emergency departments.^35,36^

In writing this paper, we draw from real-world knowledge of conducting clinical trials from three different studies, conducted on different wards across a range of geographical locations. However, as a limitation we fully accept that our top tips are based on our own anecdotal experience, so we may be mistaken in thinking that the factors we identified were in fact responsible for effective recruitment and retention. We may also have been unaware of other factors that actually had a more significant effect. This knowledge gap on recruitment strategies of proven effectiveness has previously been highlighted by two systematic reviews of effective strategies to improve trial recruitment.^37,38^ The 2006 review by McDonald and colleagues concluded it was unclear what trial factors predicted good recruitment, based on a sample of multi-centre trials (*N* = 114).^37^ The later 2013 Cochrane Review of trials testing the effectiveness of various recruitment strategies (*N* = 45) also found a limited evidence base, and highlighted that some of the studies included in the review had in fact used hypothetical trial scenarios to evaluate various recruitment strategies, so generalisability to real-world trials was questionable. Similarly, a study on loss to follow-up in a large, multi-centre treatment trial (*N* = 1117 trial participants) concluded they could not identify any strong predictors of loss to follow-up, despite testing multiple participant, clinician, and centre variables.^39^

In future research, one way of addressing this knowledge gap would be to encourage the greater use of SWATs (studies within trials) as routine practice in psychiatric in-patient trials. A SWAT is defined as a ‘self-contained research study that has been embedded within a host trial with the aim of evaluating or exploring alternative ways of delivering or organising a particular trial process’.^40^ Making the routine inclusion of SWATs into future trials would make a significant contribution to the evidence base on what strategies are effective; for example, testing if it makes a difference who makes the initial approach to a potential participant. A future systematic review could also address the question of how recruitment and retention rates for psychology in-patient trials may be changing over time, following a similar methodology to the Walters et al review of NIHR-funded trials.^1^

Ensuring good recruitment and retention for RCTs conducted on psychiatric wards is important to reduce research waste, and to further the evidence base on the effectiveness of psychological interventions within these uniquely challenging settings. When planning and designing future in-patient trials, research teams should aim to draw both on literature from the wider trials evidence base where relevant, in addition to the in-patient-specific literature.

## Data Availability

Data availability is not applicable to this article, as no new data were created or analysed in this study.
